# Nitrate leaching in a winter wheat-summer maize rotation on a calcareous soil as affected by nitrogen and straw management

**DOI:** 10.1038/srep42247

**Published:** 2017-02-08

**Authors:** Tao Huang, Xiaotang Ju, Hao Yang

**Affiliations:** 1 College of Resources and Environmental Sciences, China Agricultural University, 2 Yuanmingyuan West Road, Beijing 100193, China; 2 School of Geography Science, Nanjing Normal University, Nanjing 210023, China

## Abstract

Nitrate leaching is one of the most important pathways of nitrogen (N) loss which leads to groundwater contamination or surface water eutrophication. Clarifying the rates, controlling factors and characteristics of nitrate leaching is the pre-requisite for proposing effective mitigation strategies. We investigated the effects of interactions among chemical N fertilizer, straw and manure applications on nitrogen leaching in an intensively managed calcareous Fluvo-aquic soil with winter wheat-summer maize cropping rotations on the North China Plain from October 2010 to September 2013 using ceramic suction cups and seepage water calculations based on a long-term field experiment. Annual nitrate leaching reached 38–60 kg N ha^−1^ from conventional N managements, but declined by 32–71% due to optimum N, compost manure or municipal waste treatments, respectively. Nitrate leaching concentrated in the summer maize season, and fewer leaching events with high amounts are the characteristics of nitrate leaching in this region. Overuse of chemical N fertilizers, high net mineralization and nitrification, together with predominance of rainfall in the summer season with light soil texture are the main controlling factors responsible for the high nitrate leaching loss in this soil-crop-climatic system.

Nitrate-N (NO_3_-N) leaching is a prominent process of nitrogen (N) loss in agricultural ecosystems because both nitrate-N and soil particles are negatively charged in most cases. Leached nitrate may induce groundwater contamination or surface water eutrophication to threaten human health^1^. Intensively managed croplands are the most important source of leached nitrate^2^,^3^. Clarifying the rates, controlling factors and characteristics of the nitrate leaching in specific soil-climate and management practices can increase our knowledge to formulate targeted mitigation strategies.

Inorganic and/or organic N input consider as the main factor controlling the nitrate leaching rate, with the higher rates usually resulting from over N inputs especially when the N inputs exceed crop demand^4^,^5^,^6^. For example, the nitrate leaching could reach to 182–277 kg N ha^−1^ due to excessive fertilization and irrigation in greenhouse vegetable systems in south China^7^. Soil and climatic conditions are also important factors controlling nitrate leaching. More nitrate leached from sandy soils (87 kg N ha^−1^) than from a loess loam soil (10 kg N ha^−1^) despite similar N application rates (213 vs. 209 kg N ha^−1^) in north Germany^8^. Soil drainage, depending mainly on precipitation/irrigation rates, also plays a key role in nitrate leaching. For instance, the irrigation rate decreased from 500 mm to no irrigation when drainage declined from 570 to 79 mm^9^. Leaching depth, nitrate concentration and nitrate leaching rate increased 3.1, 1.9 and 6.8 times, respectively, when the annual rainfall increased from 185 to 318 mm^10^. In summary, nitrate leaching is greatly influenced by edaphic and climatic factors and agricultural management practices. Local nitrate leaching characteristics are specifically determined by these factors and also their interactions^11^.

The intensive double-cropping system with flood irrigated winter wheat and rain-fed summer maize rotations mainly practiced in a cereal cultivated area of 30 million ha in the North China Plain (NCP), which contributes 48 and 39% of the total wheat and maize production in China, respectively. Farmers in this region usually irrigate with large amounts of water and apply large amounts of fertilizer N to obtain relatively high yields. These practices lead to substantial accumulation of nitrate in the soil profile^12^,^13^. The residual nitrate easily leached down to deeper soil layers during the summer maize growing season with heavy rainfall events^5^,^9^,^14^. The characteristics of nitrate leaching in this combination of soil and climate are therefore unique and significantly different from other regions of the world, especially the winter nitrate leaching in Europe^15^,^16^.

Previous studies have investigated nitrate leaching on the NCP under various forms of agricultural management practices. Little or no nitrate leaching was found in the winter wheat season due to a lack of drainage but leaching occurred during the heavy precipitation of the summer maize season^17^. Rainfall and irrigation rates were found to play key roles in nitrate leaching using a Br^−^ tracer method and HYDRUS-1D model^9^. The nitrate leaching rate increased from 14.6 to 177.8 kg N ha^−1^ as the N application rate increased (0–720 kg N ha^−1^)^5^,^14^. However, fewer studies analyze the characteristics of nitrate leaching or integrate the soil-climatic and agricultural management factors in this region.

Lysimeters are widely used to quantify nitrate leaching *in situ* because they can quantify the nitrate concentration and volume of water flow directly^4^,^18^. Leachate collected by this method can reflect the actual situation of field management, but has disadvantages in terms of high soil disturbance, high cost, and long-term to stable and sidewall flow^19^. It may also underestimate the nitrate leaching if the soils are unsaturated^18^. Nitrate leaching can be determined by multiplying the nitrate concentration in soil solution (collected with porous cups) with the drainage volume (calculated by equations and models)^16^,^20^. The porous cups method are convenient and have been used widely to evaluate nitrate leaching on the NCP because they involve less soil disturbance and easy installation^5^,^21^,^22^.

The objectives of the present study were to quantify the effects of N fertilization by improved N_min_ test (optimum N) and balance calculation methods on crop yields, N surplus, soil nitrate accumulation, and nitrate leaching; to clarify the characteristics of nitrate leaching in this specific soil-climatic context with winter wheat-summer maize double cropping systems; to explore the effects of interactions among chemical N fertilizer, straw and compost applications on nitrate leaching; and to investigate the relationships between nitrate leaching rate and N surplus or water supply.

## Results

### N input, grain yield and aboveground N uptake

Compared to the conventional N treatments (N_con_ & N_con_+S), the optimum N (N_opt_ & N_opt_+S) saved 18–64% fertilizer N without significantly grain yield decreasing (Tables 1 and 2 and S1). The compost manure or municipal waste treatments (M_bal_+S & W_bal_+S) saved 40–85% chemical fertilizer N with 2–36% grain yield increases, compared to the conventional treatments (N_con_ & N_con_+S) (Tables 1 and 2). The crop yield and N uptake in the compost treatments was the highest across the years. The annual grain yield and N uptake from straw return treatments did not increase significantly over the three years, compared to the straw removal treatments (Table 2 and S1).

### Soil NO_3_-N accumulation at 0–1 m and 1–2 m depths

The amount of NO_3_-N accumulation at 0–1 m and 1–2 m depth varied greatly among treatments, years and crops (Tables S2 and S3). The highest amounts were 896 and 873 kg N ha^−1^ from N_con_ at 0–1 m and 1–2 m depth, respectively. The optimum N treatment (N_opt_) reduced the amount by 51–86 and 60–73% at 0–1 m and 1–2 m depth, respectively, relative to the conventional treatments (N_con_) (*P* < 0.05). There were no significantly differences (*P* > 0.05) between the straw removal and straw-return treatments at 0–1 m or 1–2 m soil depth. There were also no significantly differences (*P* > 0.05) between N_opt_+S and compost treatments (M_bal_+S & W_bal_+S) at either soil depth.

The distribution of NO_3_-N at the 0–2 m soil depth showed pronounced seasonal variation (Fig. S1). There were no clear NO_3_-N accumulated peaks from any soil depths in the control treatments (N_0_ & N_0_+S). In all N treatments, there were some small NO_3_-N accumulation peaks at 20–80 cm soil depth after the winter wheat season, which increased to 120–180 cm soil depth in the summer maize season. The trends in NO_3_-N accumulation peaks in the wheat season moving from shallow (above 1 m) to deep (below 1 m) in the maize season were more rapid and pronounced, especially in 2011 and 2012 (Figs S1 and S2).

### N balance and N surplus

The study soil had a high apparent N mineralization capacity which ranged from 40 to 98 and 73 to 115 kg N ha^−1^ in the wheat and maize seasons, respectively (Table S4). The annual apparent N losses were in the order: N_con_ (281 kg N ha^−1^) > N_con_+S (272 kg N ha^−1^) > W_bal_+S (84 kg N ha^−1^) > N_opt_ (80 kg N ha^−1^) > N_opt_+S (70 kg N ha^−1^) > M_bal_+S (63 kg N ha^−1^).

The N surpluses from October 2010 to September 2013 are shown in Table 3. All annual N surpluses from different treatments were in the order: controls (−175 to −121 kg N ha^−1^) < optimum treatments (−29 to 14 kg N ha^−1^) < compost treatments (71 to 89 kg N ha^−1^) < conventional treatments (183 to 282 kg N ha^−1^). The optimum N treatments almost maintained a balance between N inputs and outputs. The highest N surpluses occurred in the conventional N treatments because of the relatively high chemical fertilizer N inputs with relatively low N uptake (Table 1 and S1). The annual N surpluses from compost treatments (M_bal_+S & W_bal_+S) were 5.1–6.4 times higher than optimum N (N_opt_ & N_opt_+S) but decreased significantly by 51.4–74.8% compared to the conventional treatment (N_con_ & N_con_+S) (Table 3).

### Nitrate concentrations and nitrate leaching losses

The patterns of nitrate concentrations in soil water samples from January 2011 to December 2013 are shown in Fig. S3. The soil water from one, two, and three replications were occupied by 24–35, 38–62, and 37–85% of total 63 soil water samples, respectively, over our measurement period. The nitrate concentrations of all plots varied from 0.3–405.7 mg NO_3_-N L^−1^, and about two thirds of seepages sampled in the maize season. The highest nitrate concentrations amounted to 230.1, 145.8, and 405.7 mg NO_3_-N L^−1^ from the optimum N (N_opt_ & N_opt_+S), compost N (M_bal_+S & W_bal_+S), and conventional N (N_con_ & N_con_+S) treatments, respectively.

The nitrate leaching over three years were in the order: conventional N (23.3–51.0 kg N ha^−1^) > compost N (10.3–31.9 kg N ha^−1^) > optimum N (12.8–25.4 kg N ha^−1^) > control (1.7–3.7 kg N ha^−1^) (Table 4). It occurred mainly in the summer maize season, which occupied by 90–98, 76–91 and 83–89% from 2011, 2012 and 2013, respectively. Compared with control, application N significantly (*P* < 0.05) increased nitrate leaching by 6.7–29.0 times (Table S1). The nitrate leaching from straw-return treatments was not significantly different from straw removal (*P* > 0.05).

There was a relationship between the nitrate leaching rate (NL) and the total N inputs (TNI) and rainfall plus irrigation rate(RI) by multi-factor stepwise regression (NL = 0.054TNI + 0.229RI − 204.446, R^2^ = 0.79, P < 0.01). This equation suggests that precipitation plus irrigation was the main factor controlling nitrate leaching in studied conditions; 79% of the variation was explained by total N inputs and precipitation plus irrigation.

### Correlations between N input, N surplus, nitrate accumulation and nitrate leaching

**Figure 1:**
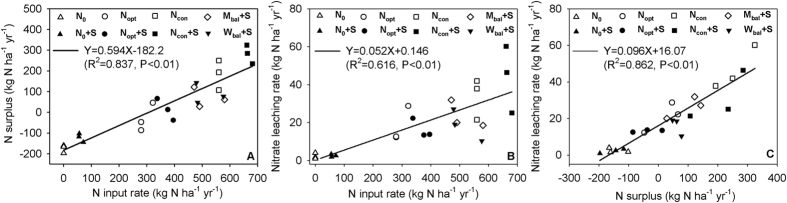
Correlations between N input and N surplus (**A**), N input and nitrate leaching rate (**B**), N surplus and nitrate leaching rate (**C**) at 1 m soil depth from 2011 to 2013.

The N input rates ranged from 0 to 681 kg N ha^−1^ yr^−1^, with the corresponding figures for N surpluses and nitrate leaching rates ranging from −197 to 325 kg N ha^−1^ yr^−1^ and 0.9 to 60.2 kg N ha^−1^ yr^−1^, respectively. Linear equations can describe the relationships well between the N input rate (fertilizer N+ compost N+ straw N) and N surplus (R^2^ = 0.837, Fig. 1A), N input rate and nitrate leaching rate (R^2^ = 0.616, Fig. 1B), and N surplus and nitrate leaching rate (R^2^ = 0.862, Fig. 1C). The amounts of nitrate accumulation at 0–1 and 1–2 m soil depths both increased exponentially with increasing N input rate (R^2^ = 0.793 & R^2^ = 0.704, Fig. S4A and B). From the equations the 0–1 and 1–2 m soil depths accumulated 41 and 55 kg N ha^−1^ yr^−1^ as nitrate if there was no N applied. Similarly, the nitrate accumulation at both 0–1 and 1–2 m soil depths increased exponentially with N surplus (R^2^ = 0.494 & R^2^ = 0.698, Fig. S4C and D). The nitrate leaching rate increased significantly with nitrate accumulation at 0–1 m (R^2^ = 0.318, P < 0.01, Fig. S4E) and 1–2 m (R^2^ = 0.592, P < 0.01, Fig. S4F) soil depth. We analyzed the correlations between the annual nitrate leaching from 2011 to 2013 and the accumulated nitrate rates from 2010 to 2012 at 0–1 m soil depth and found a significant positive relationship (R^2^ = 0.318, P < 0.01) (Fig. S4E). It suggests that 4.3% of the accumulated nitrate from the previous crop harvest at 0–1 m soil depth could leach in the following year.

### Correlations between monthly precipitation plus irrigation and nitrate leaching

**Figure 2:**
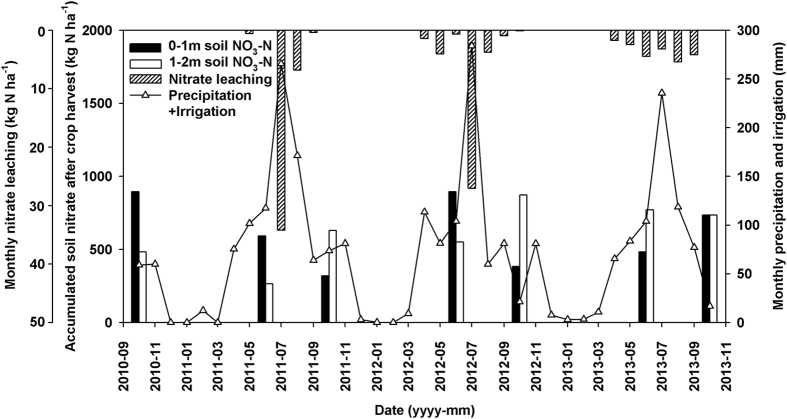
Monthly nitrate leaching rate, accumulated soil nitrate after crop harvest at 0–1 and 1–2 m depths, and monthly precipitation and irrigation in the conventional N treatments (average of N_con_ and N_con_+S) from October 2010 to September 2013.

**Figure 3:**
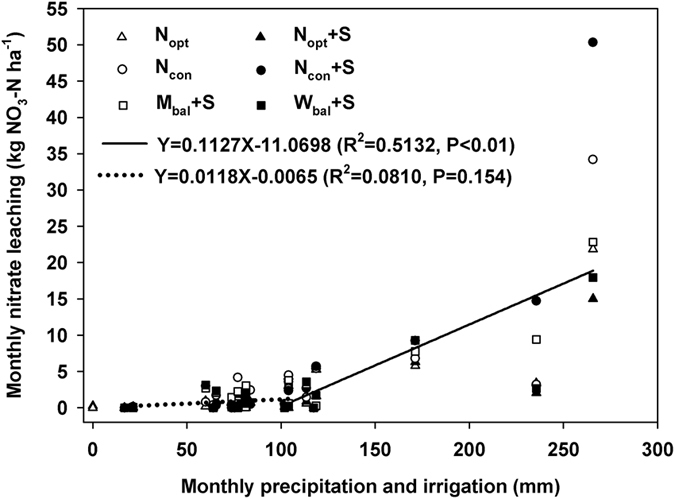
Regression lines between monthly precipitation plus irrigation and monthly nitrate leaching rate at 1 m soil depth from 2011 to 2013.

We further analyzed the relationships between the nitrate leaching rate and water supply in terms of precipitation plus irrigation. Higher monthly precipitation plus irrigation coincided with the highest monthly nitrate leaching in June 2011 and 2012 from the conventional N (N_con_ & N_con_+S), respectively, but not on June 2013 (Fig. 2). The changes in nitrate accumulation at 0–1 and 1–2 m soil depths from the winter wheat harvest to the summer maize harvest matched well with the highest nitrate leaching events in 2011 and 2012. However, the nitrate leaching rate in June 2013 was low and the 0–1 m depth cumulative soil nitrate increased by 243 kg N ha^−1^ although there was also higher monthly precipitation plus irrigation (103 mm), likely due to higher aboveground N uptake (Table S1) and evenly-distributed precipitation (Fig. S2). We found that the monthly nitrate leaching increased significantly when the monthly precipitation and irrigation amounted to over 98.2 mm (Fig. 3).

## Discussion

### Effects of N input and N surplus on nitrate leaching

The annual grain yield and N uptake were mostly not significantly different between optimized N and conventional N, but the nitrate leaching rates were significantly reduced (12.2–28.7 vs. 37.8–60.2 kg N ha^−1^) (Tables 2 and 4 & S1). A previous study in this region also shows that the nitrate leaching decreased from 177.8 to 52.5 kg N ha^−1^ yr^−1^ if the N rate was halved (from 720 to 360 kg N ha^−1^ yr^−1^)^14^. Li *et al*.^5^ indicate that the annual nitrate leaching decreased sharply from 149 to 6 kg N ha^−1^ if the applied N rate was reduced from 800 to 200 kg N ha^−1^. A recent meta-analysis shows the nitrate leaching was reduced by 40% if the recommended fertilizer rate was applied to match crop N demand^3^. Consequently, the overuse of chemical N fertilizer, higher than the amount of N taken up by the crops, is the key factor responsible for high nitrate leaching in this region.

Nowadays, farmers are recommended to return straw to the field to maintain soil fertility because of a switch to coal or natural gas for household fuel^23^. A previous study shows that straw incorporation can decrease nitrate leaching loss because it reduces net soil mineralization^24^. The high C/N ratio of straw incorporated into the soil can transform mineral N to organic N by immobilization^12^.

Our results showed that fertilizer N combining with compost N did not significantly increase nitrate leaching (P > 0.05) (Table 4). A previous study of a maize-alfalfa system in the US indicates that manure applications require careful management because the highest nitrate leaching losses occurred in the manure treatments^4^. The N surplus and nitrate leaching rates from the compost N (M_bal_+S & W_bal_+S) were higher than optimum N (N_opt_+S) mainly because we only considered 60% of the total Kjeldahl N from the compost manure or municipal waste in our N balance calculation. Thus, we need to fully consider available N into total N inputs because late season mineralization occurs when compost is included in the fertilization regime.

Nitrogen surplus is a common indictor used to reflect the risk of nitrate leaching on a field scale^25^. A recent summary of field experimental datasets reports that N surplus and nitrate leaching can be described using exponential models (R^2^ = 0.28, P < 0.01 and R^2^ = 0.55, P < 0.01) from Chinese wheat and maize cropping systems, respectively^26^. Our results show that the linear correlation between N surplus and nitrate leaching rate at 0–1 m soil depth is highly significant and positive (R^2^ = 0.862, P < 0.01) (Fig. 1C). A multi-year study in the Po Valley in Italy reveals that N surplus and nitrate leaching also had significant linear relationship (R^2^ = 0.89, P < 0.01)^16^. Our results show that the N surpluses (107–325 kg N ha^−1^ yr^−1^) were far higher than nitrate leaching rate (21.4–60.2 kg N ha^−1^ yr^−1^) in the conventional N, indicating that there are other N pathways (ammonia volatilization, NO_3_-N accumulation) in this soil-crop-climate system. Previous studies have shown higher NH_3_ volatilization losses (about 19.4–24.7 kg N ha^−1^ yr^−1^), lower denitrification (about 0.1–3.3% of applied N) and high net mineralization potential generally on the NCP^11^,^12^,^27^. Furthermore, we found that the nitrate leaching rate was up to 16 kg N ha^−1^ yr^−1^ although the N surplus and N input rates were 0 and 306 kg N ha^−1^ yr^−1^, respectively, accounting for 5.2% of N input rates (Fig. 1).

### Characteristics of nitrate leaching in winter wheat-summer maize on the NCP

Nitrate is carried by soil water flow and can lead to leaching loss if there is enough movement of water out of the root zone. Nitrate leaching always occurs during the drainage season when precipitation and/or irrigation are higher than evaporation^13^,^28^. Recent multiple site and year research in Denmark reveals that both the nitrate leaching rates and the nitrate concentrations increase with increasing precipitation^29^. In our study site, the mean annual precipitation was 691 mm, with 72–83% of the rainfall occurring in the summer maize season (June to September) (Fig. S2) and soil water seepage occurred. A previous lysimeter field study in the same region shows that leachate volumes and nitrate leaching rate were only 4–13 mm and 0.9–13.3 kg NO_3_-N ha^−1^ in the winter wheat season^14^. We determined that the proportion of nitrate leaching in the summer maize season (83–99%) was far higher than in the winter wheat season (1–17%) on an annual basis (Table 4). However, many European studies show that nitrate leaching events occur mostly in winter and spring, mainly because these countries have temperate marine or Mediterranean climates with relatively hot dry summers and mild moist winters^16^,^30^,^31^.

Our soil core study shows that nitrate accumulated to 385–762 and 294–892 kg N ha^−1^ at 0–1 and 1–2 m soil depths in the conventional treatments (Tables S2 and S3; Fig. S1). This high accumulation of nitrate is seldom found in European soils. For example, only 15–41 kg N ha^−1^ was reported in Sweden (0–60 cm)^31^, 49–124 kg N ha^−1^ in Italy (0–140 cm)^32^ and 19–174 kg N ha^−1^ in Germany (0–140 cm)^33^. The accumulated nitrate is prone to occasional leaching under specific conditions such as heavy rainfall events. For example, two excessive rainfall events occurred on June 23^rd^ 2011 (112 mm) and July 21^st^ 2012 (165 mm) and these coincided with two periods of high soil drainage (Figs S2 and S5). The nitrate leaching rates at these two events were 10.9–18.1 and 5.5–17.6 kg N ha^−1^, representing 35.5–43.5 and 29.7–41.8% of the annual nitrate leaching rate. Similarly, a water balance study in the same region only found three or four notable soil water seepage events at 180 cm depth in a year^5^. Another study in an area with similar rainfall pattern showed only 15 nitrate leaching events during a five-year lysimeter study on Guanzhong Plain in northwest China^34^. Therefore, fewer nitrate leaching events with high single leaching rate are another important feature of nitrate leaching in the studied region.

The annual nitrate leaching in 2013 (10.3–25.1 kg N ha^−1^) was lower than that in 2011 (22.2–60.2 kg N ha^−1^) or 2012 (12.2–46.4 kg N ha^−1^) although irrigation and rainfall were not low (859 vs. 891 and 927 mm) (Table 4 and Fig. S2), because the nitrate leaching rates were not only affected by the total amounts of irrigation and rainfall, but also affected by the intensity of single rainfall events^35^. For instance, there were 3–5 events with over 50 mm rainfall in the 2011 and 2012 maize seasons, but this thing did not occur during the 2013 maize season (Figs S2 and S5). Our soil core study shows that the peaks of NO_3_-N in conventional N treatments (N_con_ & N_con_+S) in the 2012 winter wheat at 0–1 m depth had moved to the 1–2 m soil depth in the 2012 summer maize season due to heavy precipitation (160 mm) during this summer season (Fig. S2). The nitrate accumulation at 0–1 m soil depth decreased and at 1–2 m soil depth increased, coinciding well with the nitrate leaching losses in the 2011 and 2012 summer maize seasons (Fig. S1). In contrast, the 0–1 m depth nitrate accumulation increased after the summer maize in 2013 but the nitrate accumulation at 1–2 m soil depth remained unchanged, suggesting that soil nitrate continued to accumulate at 0–1 m depth. This is partly supported by Ju *et al*.^13^ who reported that the soil profile nitrate showed the characteristics of long term accumulation and occasional movement on the NCP. In addition, the increase in annual aboveground N uptake from 2012 (372 kg N ha^−1^) to 2013 (450 kg N ha^−1^) (Table S1) also played a role in decreasing nitrate leaching in 2013^5^,^7^.

Both laboratory and field studies have shown that higher N mineralization and nitrification, lower N immobilization and lower denitrification were the prominent features of calcareous Flvo-aquic soil on the NCP^36^,^37^. We further indicate that this soil type has higher nitrate leaching potential because the nitrate accumulation at 0–1 and 1–2 m soil depths remained very high (62–318 kg N ha^−1^) (Tables S2 and S3) although the annual N surplus was almost in balance (−86–66 kg N ha^−1^) in the optimized N (N_opt_ & N_opt_+S) (Table 3). In addition, this soil has a light texture with lower clay content which is favorable to increase water permeability^9^. Therefore, higher nitrification and lower denitrification with light soil texture is another feature contributing to high nitrate leaching in this soil-crop-climate system. Increasing soil N immobilization and reducing soil N nitrification to avoid excessive nitrate accumulation in the soil profile should be effectively measure to reduce nitrate leaching loss.

### Effects of precipitation and irrigation on nitrate leaching

It is well known that water supply is the most important factor controlling soil drainage and influences nitrate leaching^9^. Precipitation during the summer maize season comprised 80.1, 75.1 and 67.6% of the 2011, 2012 and 2013 annual precipitation (Fig. S2), and 90–98, 76–91 and 83–89% nitrate leaching correspondingly occurred in the 2011, 2012 and 2013 summer maize seasons. Nitrate leaching was also significantly affected by the intensity of single rainfall events as discussed above. Nitrate concentration in the soil solution should be diluted if soil drainage increases and this might control the rate of nitrate leaching. However, our results agree with previous studies showing that the total water volume plays a crucial role in explaining variations in nitrate leaching rate using current methods^24^,^29^ (Fig. S3). Therefore, the characteristics of precipitation and irrigation can strongly influence the nitrate leaching rates in this soil-crop-climate system. Optimized irrigation management such as increasing irrigation frequency and/or duration and decreasing the irrigation rate can effectively reduce nitrate leaching^9^.

We may have underestimated nitrate leaching to some extent due to measuring limitations and failure to obtain a soil water sample in every replicate suction cup. Grossmann and Udluft^19^ indicate that this technique would underestimate nitrate leaching unless there is sufficient replication. Although we did not measure nitrate leaching during the winter (December to March) due to frozen conditions (when the temperature was <0 °C because use of tensiometers requires liquid water to read the scale), this may have led to small errors due to lower soil seepage in the winter. A lysimeter field study in the same region shows that leachate volumes and nitrate leaching rate were only 4–13 mm and 0.9–13.3 kg NO_3_-N ha^−1^ over a whole winter wheat season (from the beginning of October to June of the following year), but 72–87 mm and 16.1–86.4 kg NO_3_-N ha^−1^ in the summer maize season (from mid-June to the end of September)^14^. Therefore, these uncertainties didn’t affect the main conclusions of our study.

## Methods

### Study site

The field experiment started in October 2006 and is located at the China Agricultural University Shangzhuang Research Station (39°48′N, 116°28′E) in suburban Beijing. The nitrate leaching measurement was from October 2010 to September 2013 in this study. The site is at an altitude of around 40 m with a typical continental monsoon climate. From 1951 to 2010 the mean annual temperature was 12.5 °C, ranging from 35 °C to −7 °C, and the mean annual precipitation was 588.1 mm of which about 70% occurred during the summer season (June to September). The air temperature, precipitation, and irrigation from October 2010 to October 2013 are shown in Fig. S2, and the physical properties of the calcareous Fluvo-aquic soil are shown in Table S5. The top 20 cm of the soil sampled to determine the basic properties in September 2006. The soil has a pH of 8.1 (soil-to-water ratio, 1:2.5), an organic carbon content of 7.1 g kg^−1^, a total N of 0.8 g kg^−1^, NO_3_-N 24.5 mg kg^−1^, NH_4_-N 1.20 mg kg^−1^, Olsen-P 7.8 mg kg^−1^, and available K 76.2 mg kg^−1^. Soil organic carbon, total N, nitrate and ammonium in different treatments at 0–20 cm soil depth before the 2010 winter wheat was sown (before the nitrate leaching monitoring) are shown in Table S6.

### Experimental design

Eight treatments were set in this experiment: N_0_ and N_0_+S (Zero N application, wheat and maize straw removed or returned, respectively); N_con_ and N_con_+S(Conventional farming practice with chemical fertilizer N application, wheat and maize straw removed or returned, respectively); N_opt_ and N_opt_+S (Chemical fertilizer N application according to improved N_min_ test, wheat and maize straw removed or returned, respectively); M_bal_+S (Composted cattle manure with chemical fertilizer N based on N balance calculation, wheat and maize straw returned); W_bal_+S (Composted municipal waste with chemical fertilizer N according to N balance calculation, wheat and maize straw returned) (Table 5). The design was a completely randomized block with three replicates and each plot area was 64 m^2^ (8 m × 8 m). Winter wheat was sown at the beginning of October and harvested in the middle of the June of the following year, and summer maize was sown subsequently and harvested at the end of September. The type of the chemical N fertilizer was urea. Previous publications give detailed information about the long-term field experiment and treatments^38^,^39^. Detailed field and crop management, soil and plant analysis can be seen in Supplementary Information (SI).

### Fertilization regime

Before 2011 the N rates were determined according to the synchronization of crop N demand and soil N supply in N_opt_+S and N_opt_ (i.e., the target crop N demand minus NO_3_-N in the root zone), the so-called improved N_min_ method. The target crop N demands for basal application and top-dressing were 100 and 200 kg N ha^−1^ at the 0–40 cm and 0–100 cm root zone depths, respectively. For summer maize the target crop N demands for N_opt_ and N_opt_+S were 100 and 160 kg N ha^−1^ of top-dressing at the four- and ten-leaf stages, with corresponding root zone depths of 0–60 and 0–100 cm as recommended^38^,^39^. In M_bal_+S and W_bal_+S the fertilizer rate was based on the N balance calculation with N output minus N input over the whole crop growing season with one-third and two-thirds, respectively, of the N applied as a basal and topdressing applications. The N output included total N uptake by the aboveground parts of wheat and the target residual NO_3_-N in the root zone (0–100 cm) after the wheat harvest. Total N uptake was assumed to be 180 kg N ha^−1^ in this region and target residual NO_3_-N was assumed to be 100 kg N ha^−1^. The N inputs contained an assumed 40% of total Kjeldahl N as available N in the organic treatments in the wheat season and residual NO_3_-N in the 0–100 cm root zone before sowing of wheat. For summer maize the difference from N output minus N input was divided into two halves for the four and ten leaf stage. Total N uptake by aboveground maize was assumed to be 160 kg N ha^−1^ and 20% post available N from organic fertilizer was added to the calculation of N input in the current maize season.

After 2011, to alleviate disturbances from preferential flow in subsurface soil as affected by frequent soil core samples, we summarized the N fertilizer application rates in the optimum N and compost treatments from 2006 to 2011. The N fertilizer application rates were changed as follows: winter wheat basal and top-dressed fertilizer N in N_opt_ and N_opt_+S were both 75 kg N ha^−1^; the summer maize in N_opt_ and N_opt_+S were 65 kg N ha^−1^ at both the four- and ten-leaf stages; in M_bal_+S and W_bal_+S, 170 kg N ha^−1^ (N uptake by the aboveground parts of wheat) minus N input (40% of total N from compost) with one-third and two-thirds of the N applied as basal and topdressed applications in the winter wheat season. For summer maize the difference from 180 kg N ha^−1^ (N uptake by the aboveground parts of maize) minus N input (20% of total N from compost) was divided into two halves for the four- and ten-leaf stages in the summer maize season.

In N_con_ and N_con_+S, the N rates followed conventional farming practice on the NCP: 150 kg N ha^−1^ as basal fertilizer followed by plowing and 150 kg N ha^−1^ at the shooting stage of wheat. For summer maize N_con_ and N_con_+S were 130 kg N ha^−1^ at both the four- and ten-leaf stages.

Phosphorus, potassium and compost were applied as basal fertilizers only for winter wheat at rates of 70 kg P ha^−1^ yr^−1^, 75 kg K ha^−1^ yr^−1^ and 30 Mg ha^−1^ yr^−1^ (fresh weight). The moisture content of the compost was determined by weighing a subsample before and after oven drying at 60 °C. The Kjeldahl N concentrations of dry solid cattle manure were 24.8, 20.9, and 13.4 g kg^−1^ (a mixture of matured forage and composted cattle manure) for the years 2010, 2011, and 2012 and corresponding values for dry municipal waste compost ware 8.7, 6.3, and 8.9 g kg^−1^.

### N balance and N surplus calculation

The N balance was calculated by apparent N mineralization^40^,^44^ and apparent N loss^41^,^45^. Soil NH_4_-N was excluded from this calculation due to its low concentration and stability throughout the crop rotation. The apparent N mineralization (N_miner_) in the control and apparent N loss (N_loss_) were calculated using the following equations:


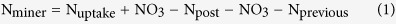



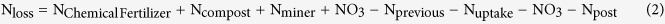


where


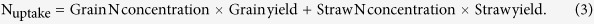


The N_miner_ is calculated in the control treatment, N_compost_ is multiplied dry weight of compost by Kjeldahl N content, which was calculated by 40% and 20% of total Kjeldahl N from winter wheat and summer maize, respectively. N_loss_ is calculated in the N application treatments, N_uptake_ is the N uptake by aboveground plant parts at crop harvest, NO_3_-N_post_ is residual NO_3_-N in 0–100 cm soil depth after current crop harvest, and NO_3_-N_previous_ is residual NO_3_-N in 0–100 cm soil depth after the previous crop harvest. The apparent N loss in straw removal treatments used the value of apparent N mineralization in the N_0_ treatment; correspondingly, straw return treatments used the value of apparent N mineralization in the N_0_+S treatment.

The N surplus was calculated using the following equation:


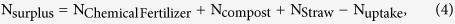


where N_Straw_ is the straw N from the last crop.

### Nitrate leaching measurement

The ceramic cups, a tensiometer, and a soil water sampler are shown schematically in Fig. S6. Soil water at 1 m soil depth was sampled using ceramic suction cups^46^,^47^ (Soil Water Sampler, Institute of Geographic Sciences and Natural Resources Research, CAS, Patent No. ZL200520110649.5). Percolation water was collected by applying a suction of 70 kPa to the cups with a vacuum hand pump, usually at 10-day intervals but more frequently immediately after irrigation, rainfall, and fertilization, except during freezes in the winter season. Leachate samples were stored in 200 ml plastic bottles and immediately frozen at −20 °C until analysis. Nitrate concentrations of the soil water were analyzed with a continuous-flow N analyzer (TRAACS 2000, Bran and Luebbe, Norderstedt, Germany). In each plot, soil-water potentials at depths of 90 cm and 110 cm were monitored at 7:00–8:00 a.m. every day with tensiometers^21^,^22^ (Tensiometer, Institute of Geographic Sciences and Natural Resources Research, CAS, Patent No. ZL200520110647.6). The measuring time was 197 days (from 21 April to 1 November), 205 days (from 3 April to 3 November), and 201 days (from 5 April to 2 November) in 2011, 2012, and 2013, respectively, and was limited by the freeze in the winter. Soil water flux at 100 cm soil depth was estimated by the soil-water potentials at 90 and 110 cm based on Darcy’s law (Fig. S5)^48^. The period of soil water flux was estimated from the sum of measurement and no-measurement days, which was estimated by linear interpolation. The nitrate leaching rate was calculated by multiplying seepage water volume for each period with the nitrate concentration of the soil water in the same period. The annual nitrate leaching rate was the sum of every period’s rate.

### Nitrate leaching Calculation

The calculated equations are as follows:


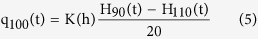



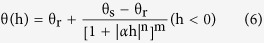



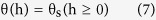



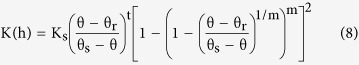



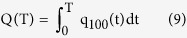



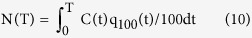


where q_100_(*t*) (cm d^−1^) is the soil water flux at 100 cm soil depth over a period of time (*t*); H_90_(*t*) and H_110_(*t*) (cm) are the soil water potentials at 90 and 110 cm soil depths over a period of time (*t*); K(*h*) (cm d^−1^) is the soil unsaturated hydraulic conductivity at 90–110 cm soil depth; *h* (cm) is the soil matrix potential; *θ* (cm^3^ cm^−3^) is the soil volumetric water content; *θ*_*r*_ and *θ*_*s*_ (cm^3 ^cm^−3^) are the residual soil water content and saturated soil water content; *θ*_*r*_, *θ*_*s*_, *α, m,* and *n* are the parameters fitted by the soil water characteristic curves at a soil depth of 90–110 cm^48^, with values of 0.01, 0.59, 0.055, 1.24, and 0.196, respectively; *K*_*s*_ (cm d^−1^) is the soil saturated hydraulic conductivity, with a value of 4.31; *Q(T)* (cm d^−1^) is the soil water flux at 100 cm soil depth over a measured period of time (*T*) (d); C(*t*) (mg L^−1^) is the nitrate concentration of the soil water at 100 cm depth; and N(*T*) (kg ha^−1^) is the nitrate leaching rate at 100 cm soil depth over a measured period of time.

### Statistical analyses

The grain yield, aboveground N uptake, soil NO_3_-N accumulation at 0–1 and 1–2 m, and nitrate leaching rate of the different treatments were tested by analysis of variance, and mean values were compared by least significant difference (LSD) at the 5% level using the SAS statistical software package (Version 8.2; SAS Institute, Inc., Cary, NC). The correlation between N inputs (chemical fertilizer N, straw N and compost N) and N surplus, N inputs and nitrate leaching rate, N surplus and nitrate leaching rate, monthly precipitation plus irrigation and monthly nitrate leaching, N inputs and soil NO_3_-N at 0–1 m, N inputs and soil NO_3_-N at 1–2 m, N surplus and soil NO_3_-N at 0–1 m, N surplus and soil NO_3_-N at 1–2 m, soil NO_3_-N at 0–1 m and nitrate leaching rate, soil NO_3_-N at 1–2 m and nitrate leaching rate were determined using SAS 8.2 Proc Mixed (SAS Institute, Inc., Cary, NC). The correlations between nitrate leaching rate and its controlling factors were analyzed by stepwise multiple linear regression.

## Additional Information

**How to cite this article**: Huang, T. *et al*. Nitrate leaching in a winter wheat-summer maize rotation on a calcareous soil as affected by nitrogen and straw management. *Sci. Rep.* **7**, 42247; doi: 10.1038/srep42247 (2017).

**Publisher's note:** Springer Nature remains neutral with regard to jurisdictional claims in published maps and institutional affiliations.

## Supplementary Material

Supplementary Information

## Figures and Tables

**Table 1:** Nitrogen rates of chemical fertilizer, compost and straw (kg N ha^−1^) in winter wheat and summer maize from October 2010 to September 2013.

Treatment#	Winter wheat				Summer maize				Annual			
	2011	2012	2013	Average	2011	2012	2013	Average	2010–2011	2011–2012	2012–2013	Average
N_0_	0(0)§	0(0)	0(0)	0(0)	0(0)	0(0)	0(0)	0(0)	0(0)	0(0)	0(0)	0(0)
N_0_+S	0(41)	0(32)	0(47)	0(40)	0(15)	0(23)	0(24)	0(21)	0(56)	0(54)	0(71)	0(61)
N_opt_	110(0)	150(0)	150(0)	137(0)	212(0)	130(0)	130(0)	157(0)	322(0)	280(0)	280(0)	294(0)
N_opt_+S	107(49)	150(44)	150(60)	136(51)	159(23)	130(52)	130(56)	140(44)	266(72)	280(96)	280(115)	275(94)
N_con_	300(0)	300(0)	300(0)	300(0)	260(0)	260(0)	260(0)	260(0)	560(0)	560(0)	560(0)	560(0)
N_con_+S	300(62)	300(47)	300(64)	300(58)	260(39)	260(56)	260(57)	260(50)	560(101)	560(103)	560(121)	560(108)
M_bal_+S	103(194)	46(201)	98(212)	82(203)	82(92)	118(125)	144(127)	115(115)	185(286)	164(327)	242(339)	197(317)
W_bal_+S	108(155)	138(103)	110(198)	119(152)	134(82)	157(86)	150(119)	147(96)	242(237)	295(189)	260(317)	266(247)

^#^N_0_, N_opt_, N_con_, M_bal_ and W_bal_ represent control, improved N_min_ test, conventional farming practice, cattle manure with N balance method and waste compost with N balance method, respectively. S represents straw returning.

^§^The number in the brackets is the sum N of compost or/and straw N, compost N was calculated by 40% and 20% of total Kjeldahl N for winter wheat and summer maize season, respectively.

**Table 2:** Grain yield (dry matter, Mg ha^−1^) of each crop from October 2010 to September 2013.

Treatment#		Winter wheat			Summer maize			Annual yield		
Nitrogen	Straw	2011	2012	2013	2011	2012	2013	2011	2012	2013
Treatment effect (n = 3)										
N_0_	S	2.7 ± 0.4§b¶	2.4 ± 0.3b	2.5 ± 0.1b	5.4 ± 0.4b	3.9 ± 0.4c	4.8 ± 0.3c	8.1 ± 0.4c	6.3 ± 0.3b	7.3 ± 0.2c
N_0_		2.9 ± 0.4b	2.5 ± 0.4b	2.7 ± 0.4b	5.0 ± 0.3b	4.5 ± 0.6c	5.0 ± 0.3c	7.9 ± 0.5c	7.0 ± 0.6b	7.7 ± 0.2c
N_opt_	S	5.4 ± 0.6a	4.3 ± 0.6a	4.5 ± 0.3a	6.5 ± 0.4ab	6.1 ± 0.3b	5.9 ± 0.4b	11.9 ± 0.5ab	10.4 ± 0.5a	10.5 ± 0.4b
N_opt_		5.4 ± 0.2a	4.7 ± 0.4a	4.7 ± 0.2a	5.9 ± 0.6b	6.5 ± 0.4ab	7.0 ± 0.3a	11.3 ± 0.7b	11.9 ± 0.7a	11.7 ± 0.2a
N_con_	S	5.5 ± 0.1a	4.5 ± 0.4a	4.7 ± 0.1a	6.5 ± 0.3ab	7.3 ± 0.4a	7.0 ± 0.2a	12.0 ± 0.3ab	11.8 ± 0.4a	11.7 ± 0.2a
N_con_		5.7 ± 0.5a	4.6 ± 0.4a	4.5 ± 0.4a	7.9 ± 0.3a	7.3 ± 0.3a	7.1 ± 0.2a	13.5 ± 0.5a	11.9 ± 0.4a	11.6 ± 0.4a
Nitrogen effect (n = 6)										
N_0_mean		2.8 ± 0.3b	2.4 ± 0.3b	2.6 ± 0.2b	5.2 ± 0.2b	4.2 ± 0.5c	4.9 ± 0.1c	8.0 ± 0.3b	6.7 ± 0.8c	7.5 ± 0.3b
N_opt_mean		5.4 ± 0.3a	4.5 ± 0.2a	4.6 ± 0.2a	6.2 ± 0.5ab	6.3 ± 0.2b	6.5 ± 0.1b	11.6 ± 0.6a	10.8 ± 0.4b	11.1 ± 0.2a
N_con_mean		5.6 ± 0.4a	4.6 ± 0.4a	4.6 ± 0.1a	7.2 ± 0.4a	7.3 ± 0.3a	7.1 ± 0.4a	12.7 ± 0.7a	11.9 ± 0.7a	11.7 ± 0.2a
Straw effect (n = 9)										
Without straw		4.5 ± 0.1a	3.7 ± 0.4a	3.9 ± 0.1a	6.1 ± 0.2a	5.8 ± 0.3a	5.9 ± 0.3a	10.7 ± 0.1a	9.5 ± 0.7a	9.8 ± 0.3a
With straw		4.6 ± 0.3a	3.9 ± 0.2a	4.0 ± 0.2a	6.3 ± 0.3a	6.1 ± 0.3a	6.4 ± 0.1a	10.9 ± 0.3a	10.0 ± 0.5a	10.3 ± 0.2a
Treatment effect (n = 3)										
N_opt_	S	5.2 ± 0.2a	4.7 ± 0.4ab	4.7 ± 0.2a	5.9 ± 0.6c	6.5 ± 0.4b	7.0 ± 0.3b	11.3 ± 0.7b	11.9 ± 0.7b	11.7 ± 0.2b
M_bal_	S	5.4 ± 0.5a	5.3 ± 0.4a	4.8 ± 0.5a	9.4 ± 0.6a	9.9 ± 0.7a	8.2 ± 0.4a	14.6 ± 0.6a	15.3 ± 0.7a	13.1 ± 0.2a
W_bal_	S	5.7 ± 0.3a	5.3 ± 0.5ab	4.7 ± 0.1a	7.7 ± 0.5b	9.2 ± 0.6a	7.9 ± 0.3a	13.4 ± 0.5ab	14.5 ± 0.6a	12.7 ± 0.9ab

^#^N_0_, N_opt_, N_con_, M_bal_ and W_bal_ represent control, improved N_min_ test, conventional farming practice, cattle manure with N balance method and waste compost with N balance method, respectively. S represents straw returning.

^§^Number represents mean ± standard error.

^¶^Means followed by the same letter are not significantly different (*P* < *0.05*).

**Table 3:** N surplus (kg N ha^−1^) of each crop from October 2010 to September 2013.

Treatment#	Winter wheat				Summer maize				Annual			
	2011	2012	2013	Average	2011	2012	2013	Average	2010–2011	2011–2012	2012–2013	Average
N_0_	−79§	−77	−82	−79	−82	−89	−115	−95	−161	−166	−197	−175
N_0_+S	−41	−43	−44	−43	−61	−74	−100	−78	−102	−117	−144	−121
N_opt_	−48	−15	−48	−37	94	−32	−38	8	46	−47	−86	−29
N_opt_+S	−1	26	−16	3	67	−13	−22	11	66	13	−38	14
N_con_	119	138	63	107	131	55	44	77	250	193	107	183
N_con_+S	176	174	143	164	149	111	92	117	325	285	235	282
M_bal_+S	125	51	69	81	−3	−22	−7	−11	122	29	62	71
W_bal_+S	78	50	69	65	65	−2	9	24	143	48	78	89

^#^N_0_, N_opt_, N_con_, M_bal_ and W_bal_ represent control, improved N_min_ test, conventional farming practice, cattle manure with N balance method and waste compost with N balance method, respectively. S represents straw returning.

^§^N_surplus_ = N_Chemical Fertilizer_ + N_Compost_ + N_Straw_ − N_uptake_.

**Table 4:** Nitrate leaching rate (kg N ha^−1^) of each crop at 100 cm soil depth.

Treatment#		Winter wheat			Summer maize			Annual		
Nitrogen	Straw	2011	2012	2013	2011	2012	2013	2010–2011	2011–2012	2012–2013
Treatment effect (n = 3)										
N_0_	S	0.1 ± 0.2§c¶	0.4 ± 0.5c	0.1 ± 0.1c	1.4 ± 0.3c	3.6 ± 2.5b	0.8 ± 0.6c	1.6 ± 0.2c	4.0 ± 2.7b	0.9 ± 0.7c
N_0_		0.2 ± 0.1c	0.6 ± 0.4c	0.4 ± 0.3c	1.6 ± 0.2c	2.7 ± 0.6b	2.1 ± 0.8c	1.8 ± 0.2c	3.3 ± 0.4b	2.5 ± 0.9c
N_opt_	S	1.0 ± 0.1a	1.6 ± 1.1b	1.6 ± 0.9b	27.7 ± 2.7b	10.6 ± 4.7b	10.9 ± 5.1b	28.7 ± 2.9b	12.2 ± 4.4b	12.5 ± 5.5b
N_opt_		0.8 ± 0.6a	2.2 ± 1.2b	1.6 ± 1.1b	21.3 ± 7.9b	10.2 ± 1.4b	12.1 ± 5.1b	22.2 ± 8.6b	13.4 ± 1.5b	13.7 ± 4.5b
N_con_	S	0.6 ± 0.2ab	3.9 ± 1.9a	3.2 ± 1.2a	41.4 ± 6.8a	33.9 ± 14.1a	18.3 ± 5.5ab	41.9 ± 7.0ab	37.8 ± 14.7a	21.4 ± 5.7ab
N_con_		0.9 ± 0.3a	4.4 ± 2.7a	3.2 ± 2.0a	59.3 ± 18.2a	42.0 ± 13.5a	21.9 ± 6.8a	60.2 ± 18.9a	46.4 ± 14.4a	25.1 ± 7.2a
Nitrogen effect (n = 6)										
N_0_mean		0.2 ± 0.1b	0.5 ± 0.5c	0.3 ± 0.2c	1.5 ± 0.1c	3.2 ± 1.0c	1.4 ± 0.7c	1.7 ± 0.1c	3.7 ± 1.1b	1.7 ± 0.8c
N_opt_mean		0.9 ± 0.4a	1.9 ± 1.1b	1.6 ± 1.0b	24.5 ± 4.5b	10.4 ± 2.5b	11.5 ± 4.1b	25.4 ± 4.7b	12.8 ± 2.9b	13.1 ± 4.0b
N_con_mean		0.7 ± 0.3a	4.1 ± 2.5a	3.2 ± 1.6a	50.3 ± 11.1a	38.0 ± 8.9a	20.1 ± 1.0a	51.0 ± 11.7a	42.1 ± 10.0a	23.3 ± 1.3a
Straw effect (n = 9)										
Without straw		0.6 ± 0.2a	2.0 ± 1.1a	1.6 ± 0.2a	23.5 ± 3.1a	16.1 ± 4.8a	10.0 ± 1.0a	24.1 ± 3.0a	18.0 ± 5.0a	11.6 ± 0.2a
With straw		0.6 ± 0.4a	2.4 ± 0.9a	1.7 ± 0.3a	27.4 ± 6.2a	18.3 ± 3.8a	12.0 ± 2.1a	28.0 ± 7.0a	21.0 ± 4.2a	13.8 ± 3.1a
Treatment effect (n = 3)										
N_opt_	S	0.8 ± 0.6a	2.2 ± 1.2a	1.6 ± 1.1a	21.3 ± 7.9a	10.2 ± 1.4a	12.1 ± 5.1a	22.2 ± 8.6a	13.4 ± 1.5a	13.7 ± 4.5a
M_bal_	S	1.5 ± 1.0a	2.8 ± 1.3a	2.3 ± 1.7a	30.4 ± 13.4a	17.2 ± 5.5a	16.2 ± 5.1a	31.9 ± 12.8a	20.0 ± 4.2a	18.5 ± 3.6a
W_bal_	S	1.1 ± 0.1a	1.8 ± 0.9a	1.2 ± 1.1a	27.0 ± 8.6a	17.2 ± 7.1a	9.1 ± 4.1a	27.1 ± 7.6a	19.0 ± 6.3a	10.3 ± 2.6a

^#^N_0_, N_opt_, N_con_, M_bal_ and W_bal_ represent control, improved N_min_ test, conventional farming practice, cattle manure with N balance method and waste compost with N balance method, respectively. S represents straw returning.

^§^Number represents mean ± standard error.

^¶^Means followed by the same letter are not significantly different (*P* < 0*.05*).

**Table 5:** Treatments of the field experiment.

Treatment	Nitrogen management	Straw management
N_0_	Zero N application	Wheat and maize straw removing
N_0_+S	Zero N application	Wheat straw mulching and maize straw returning
N_opt_	Improved N_min_ test	Wheat and maize straw removing
N_opt_+S	Improved N_min_ test	Wheat straw mulching and maize straw returning
N_con_	Conventional farming practice	Wheat and maize straw removing
N_con_+S	Conventional farming practice	Wheat straw mulching and maize straw returning
M_bal_+S	Composted cattle manure with chemical fertilizer N based on N balance calculation	Wheat straw mulching and maize straw returning
W_bal_+S	Composted municipal waste with chemical fertilizer N based on N balance calculation	Wheat straw mulching and maize straw returning
