# Supplementary material for: Nitrate leaching in a winter wheat-summer maize rotation on a calcareous soil as affected by nitrogen and straw management
Source: Sci Rep. 2017 Feb 8;7:42247. doi: 10.1038/srep42247 (PMC5296732; doi:10.1038/srep42247)
Supplement: Supplementary Information [file srep42247-s1.doc]

**Supplementary Information**

**Nitrate leaching in a winter wheat-summer maize rotation on a calcareous soil as affected by nitrogen and straw management**

Tao Huang ^a,b^ , Xiaotang Ju^a^*, Hao Yang^b^

*^a^ College of Resources and Environmental Sciences, China Agricultural University, 2 Yuanmingyuan West Road , Beijing 100193, China.*

*^b^ School of Geography Science, Nanjing Normal University, Nanjing 210023, China.*

*Corresponding author: Xiaotang Ju

College of Resources and Environmental Sciences, China Agricultural University, Beijing 100193, China.

Tel: +86-10-62732006; Fax: +86-10-62731016.

E-mail: [juxt@cau.edu.cn](mailto:juxt@cau.edu.cn)

Field and crop management

The row spacing of winter wheat (var. Nongda 211) was 15 cm and the sowing rate was 225 kg ha^-1^. The distances between rows and plants for summer maize (var. Zhengdan 958) were 60 and 25 cm, respectively. Chemical fertilizer, compost and crushed maize stalks were incorporated into the soil, with tractor-plowed tillage (20–25 cm depth) at the beginning of October before the wheat was sown, and the top-dressing N fertilizer was manual broadcast at the shooting stage of wheat and followed by irrigation. The fertilizer N was band-spread and the soil covered by machine before a precipitation event or supplementary irrigation at the four- and ten-leaf stages of summer maize; the wheat straw was mulched on the soil surface after the wheat harvest. Both maize stalks and wheat straws were mechanically chopped into 5–8 cm lengths. All the plants in straw-removal plots were cut down manually and removed after yield-sampling by hand.

The irrigation rate depended on the soil moisture conditions. Winter wheat was irrigated with 60, 60, 40, and 40 mm in 2010–2011; 60, 60, 0, and 50 mm in 2011–2012; and 60, 60, 60, and 0 mm in 2012–2013 before the winter freeze at sowing, shooting, heading, and grain filling, respectively. An additional 40 mm of irrigation was supplied to the winter wheat seedlings on 3 October 2011 due to a drought period. No additional irrigation was used for summer maize seedlings because rain events coincidentally occurred after the seedling stage. Pesticides (a mixture of *dichlorovos* and *dimethoate*) were sprayed in the middle of April and the middle or end of May. The same pesticide mixture supplied to wheat and herbicide (*acetochlor*) was sprayed after summer maize sowing. The liquid pesticide mixture was applied again at the beginning of July and a solid granular pesticide (*carbofuran*) was applied to the top leaves of summer maize at the beginning of August.

Soil and plant analysis

Soil samples were taken from the top 200 cm of the soil profile in each plot after harvest. Soil cores were collected from two points in each plot, separated into 20-cm depth intervals, mixed thoroughly to obtain composite samples from each depth layer, placed in labeled plastic bags, and sealed and stored frozen before analysis for NH_4_-N and NO_3_-N in the laboratory. Each fresh soil sample was sieved with a 3 mm mesh and extracted with 0.01 mol L^-1^ CaCl_2_ at a soil-to-water ratio of 1:10 (W/V) to determine the concentrations of NH_4_-N and NO_3_-N using a continuous flow analyzer (TRAACS 2000, Bran and Luebbe, Norderstedt, Germany).

At the winter wheat harvest fresh aboveground biomass was harvested from an area in the middle of each plot that measured 9 m^2^ (3× 3 m). Grain and straw samples were oven-dried at 65 °C for determination of dry matter yield. In the case of the summer maize harvest, 14.4 m^2^ (six rows 4 m in length) in the center of each plot were harvested to determine the fresh ear and stover yields as well as ear number. Five plants were randomly selected from the harvested summer maize and separated into grain, cob, and stover to determine the oven-dried weight at 65 °C. The grain yield of maize was calculated by deduction from cob yield. The C and N contents of grain and straw for both winter wheat and summer maize were determined using a CN analyzer (Vario Max CN, Elemental, Hanau, Germany).

**Figure legends**

Fig.S1 Soil NO_3_-N (kg N ha^-1^) distribution at 0–2 m soil depth after each crop harvest from October 2010 to September 2013. WW represents winter wheat. SM represents summer maize.

Fig.S2 Air temperature, precipitation and irrigation in the winter wheat-summer maize double cropping system from October 2010 to September 2013.

Fig.S3 Soil water NO_3_-N concentrations sampled with suction cups at 1 m depth from January 2011 to December 2013.

Fig. S4 Correlations between N input and nitrate accumulation at 0-1 m soil depth (A), N input and nitrate accumulation at 1-2 m soil depth (B), N surplus and nitrate accumulation at 0-1 m soil depth (C), N surplus and nitrate accumulation at 1-2 m soil depth (D), nitrate accumulation at 0-1 m soil depth and nitrate leaching rate (E), nitrate accumulation at 1-2 m soil depth and nitrate leaching rate (F).

Fig.S5 Soil water potential (cm) at 90 and 110 cm soil depths in the field experiment from April to November in 2010, 2011, and 2012.

Fig.S6 Schematic view of ceramic cups, tensiometer and soil solution sampler.

Table S1 Nitrogen uptake (kg N ha^-1^) by aboveground parts after each crop harvest from October 2010 to September2013

| Treatment^#^ | |  | Winter wheat | | |  | Summer maize | | |  | Annual | | |
| --- | --- | --- | --- | --- | --- | --- | --- | --- | --- | --- | --- | --- | --- |
| Nitrogen | Straw |  | 2011 | 2012 | 2013 |  | 2011 | 2012 | 2013 |  | 2011 | 2012 | 2013 |
| Treatment effect (n = 3) | |  |  |  |  |  |  |  |  |  |  |  |  |
| N_0_ |  |  | 79±13^§^b^¶^ | 77±12b | 82±2c |  | 82±7c | 89±9c | 115±3c |  | 161±19c | 166±11c | 197±5c |
| N_0_ | S |  | 82±20b | 75±10b | 91±15c |  | 76±9c | 97±17c | 124±11c |  | 158±28c | 171±26c | 215±26c |
| N_opt_ |  |  | 158±17a | 165±29a | 198±10b |  | 118±11b | 162±15b | 168±13b |  | 276±13b | 327±28b | 366±20b |
| N_opt_ | S |  | 157±9a | 168±15a | 226±12a |  | 115±25b | 195±5a | 208±6a |  | 272±32b | 363±18ab | 435±15a |
| N_con_ |  |  | 181±23a | 162±7a | 237±9a |  | 129±14ab | 205±17a | 216±16a |  | 309±37ab | 367±23ab | 454±24a |
| N_con_ | S |  | 186±15a | 173±7a | 221±18ab |  | 150±10a | 205±9a | 225±5a |  | 336±19a | 377±13a | 446±15a |
| Nitrogen effect (n = 6) | |  |  |  |  |  |  |  |  |  |  |  |  |
| N_0_mean | |  | 81±12b | 76±7b | 86±8b |  | 79±6c | 93±13c | 119±7c |  | 160±17c | 169±16b | 206±15c |
| N_opt_mean | |  | 157±9a | 167±14a | 212±6a |  | 126±15a | 178±3b | 188±6b |  | 284±11a | 345±13a | 400±3b |
| N_con_mean | |  | 183±15a | 167±7a | 229±12a |  | 139±3a | 205±7a | 221±7a |  | 323±18a | 372±14a | 450±20a |
| Straw effect (n = 9) | |  |  |  |  |  |  |  |  |  |  |  |  |
| Without straw | |  | 139±8a | 135±12a | 172±4a |  | 110±6a | 152±8a | 166±7a |  | 249±10a | 287±20a | 339±11a |
| With straw | |  | 142±7a | 139±7a | 179±8a |  | 114±8a | 165±6a | 186±2a |  | 255±10a | 304±13a | 365±7a |
| Treatment effect (n = 3) | |  |  |  |  |  |  |  |  |  |  |  |  |
| N_opt_ | S |  | 157±9a | 168±15a | 226±12ab |  | 115±25c | 195±5b | 208±6b |  | 272±32b | 363±18b | 435±15b |
| M_bal_ | S |  | 172±16a | 197±12a | 241±18a |  | 177±8a | 265±20a | 278±13a |  | 349±21a | 462±31a | 519±6a |
| W_bal_ | S |  | 185±5a | 191±17a | 239±11a |  | 141±14b | 245±7a | 260±15a |  | 326±10a | 436±22a | 499±21a |

^#^ N_0_, N_opt_, N_con_, M_bal_ and W_bal_ represent control, improved N_min_ test, conventional farming practice, cattle manure with N balance method and waste compost with N balance method, respectively. S represents straw return.

^§^ Number represents mean ± standard error.

^¶^ Means followed by the same letter are not significantly different (*P <0.05*).

Table S2 Soil NO_3_-N (kg N ha^-1^) accumulation in the root zone (0-1 m) after each crop harvest from October 2010 to September 2013

| Treatment^#^ | |  | Winter wheat | | |  | Summer maize | | |
| --- | --- | --- | --- | --- | --- | --- | --- | --- | --- |
| Nitrogen | Straw |  | 2011 | 2012 | 2013 |  | 2011 | 2012 | 2013 |
| Treatment effect (n = 3) | |  |  |  |  |  |  |  |  |
| N_0_ |  |  | 18±4^§^c^¶^ | 31±15c | 33±17c |  | 35±9e | 18±6d | 18±4c |
| N_0_ | S |  | 20±4c | 31±5c | 44±7c |  | 48±7de | 47±14cd | 28±19c |
| N_opt_ |  |  | 62±15b | 266±143b | 196±51b |  | 145±46bc | 151±106bc | 188±40b |
| N_opt_ | S |  | 97±21b | 194±46b | 265±98ab |  | 98±28cd | 178±81ab | 257±60b |
| N_con_ |  |  | 592±122a | 896±244a | 484±174a |  | 320±188ab | 485±221a | 735±27a |
| N_con_ | S |  | 530±153a | 627±157a | 465±68a |  | 449±193a | 384±176ab | 699±228a |
| Nitrogen effect (n = 6) | |  |  |  |  |  |  |  |  |
| N_0_mean | |  | 19±1c | 31±10c | 38±6c |  | 41±3c | 33±10c | 23±11c |
| N_opt_mean | |  | 79±18b | 230±56b | 231±32b |  | 122±25b | 165±32b | 222±25b |
| N_con_mean | |  | 561±70a | 762±275a | 475±86a |  | 385±13a | 434±165a | 717±154a |
| Straw effect (n = 9) | |  |  |  |  |  |  |  |  |
| Without straw | |  | 224±37a | 397±95a | 238±59a |  | 167±59a | 218±84a | 313±11a |
| With straw | |  | 215±48a | 284±73a | 258±27a |  | 199±53a | 203±89a | 328±109a |
| Treatment effect (n = 3) | |  |  |  |  |  |  |  |  |
| N_opt_ | S |  | 97±21a | 194±46a | 265±98a |  | 98±28a | 178±81a | 257±60a |
| M_bal_ | S |  | 137±37a | 178±92a | 210±62a |  | 242±104a | 175±42a | 393±42a |
| W_bal_ | S |  | 80±14a | 169±48a | 376±63a |  | 188±94a | 226±66a | 481±106a |

^#^ N_0_, N_opt_, N_con_, M_bal_ and W_bal_ represent control, improved N_min_ test, conventional farming practice, cattle manure with N balance method and waste compost with N balance method, respectively. S represents straw return.

^§^ Number represents mean ± standard error.

^¶^ Means followed by the same letter are not significantly different (*P <0.05*).

Table S3 Soil NO_3_-N (kg N ha^-1^) accumulation at 1–2 m soil depth after each crop harvest from October 2010 to September 2013

| Treatment^#^ | |  | Winter wheat | | |  | Summer maize | | |
| --- | --- | --- | --- | --- | --- | --- | --- | --- | --- |
| Nitrogen | Straw |  | 2011 | 2012 | 2013 |  | 2011 | 2012 | 2013 |
| Treatment effect (n = 3) | |  |  |  |  |  |  |  |  |
| N_0_ |  |  | 34±29^§^d^¶^ | 59±51c | 73±50c |  | 68±33d | 37±11c | 39±9b |
| N_0_ | S |  | 55±23cd | 99±51bc | 76±39c |  | 141±29cd | 46±34c | 59±38b |
| N_opt_ |  |  | 101±35abc | 249±102ab | 297±121b |  | 263±99bc | 245±57b | 307±61a |
| N_opt_ | S |  | 84±21bc | 135±17b | 256±37b |  | 230±43c | 232±49b | 318±116a |
| N_con_ |  |  | 265±49ab | 551±204a | 772±178a |  | 630±157ab | 873±226a | 736±196a |
| N_con_ | S |  | 323±93a | 576±183a | 619±131ab |  | 675±179a | 911±237a | 826±293a |
| Nitrogen effect (n = 6) | |  |  |  |  |  |  |  |  |
| N_0_mean | |  | 45±26c | 79±30c | 75±33c |  | 104±19c | 41±22c | 49±23b |
| N_opt_mean | |  | 92±21b | 192±52b | 277±54b |  | 247±51b | 238±7b | 313±77a |
| N_con_mean | |  | 294±65a | 563±101a | 695±60a |  | 653±14a | 892±173a | 781±99a |
| Straw effect (n = 9) | |  |  |  |  |  |  |  |  |
| Without straw | |  | 133±32a | 286±95a | 381±57a |  | 320±89a | 385±125a | 361±90a |
| With straw | |  | 154±38a | 270±72a | 317±48a |  | 349±79a | 396±161a | 401±158a |
| Treatment effect (n = 3) | |  |  |  |  |  |  |  |  |
| N_opt_ | S |  | 84±21a | 135±17a | 256±37a |  | 230±43a | 232±49a | 318±116a |
| M_bal_ | S |  | 125±50a | 202±32a | 332±99a |  | 274±145a | 231±82a | 243±52a |
| W_bal_ | S |  | 93±20a | 181±57a | 238±25a |  | 170±8a | 288±35a | 262±21a |

^#^ N_0_, N_opt_, N_con_, M_bal_ and W_bal_ represent control, improved N_min_ test, conventional farming practice, cattle manure with N balance method and waste compost with N balance method, respectively. S represents straw return.

^§^ Number represents mean ± standard error.

^¶^ Means followed by the same letter are not significantly different (*P <0.05*).

Table S4 Apparent N mineralization and N balance (kg N ha^-1^) after each crop harvest from October 2006 to September 2013

| Treatment | Wheat (n = 3) | | | | | | |  | Maize (n = 3) | | | | | | |  | Total |  | Yearly |
| --- | --- | --- | --- | --- | --- | --- | --- | --- | --- | --- | --- | --- | --- | --- | --- | --- | --- | --- | --- |
|  | 2007 | 2008 | 2009 | 2010 | 2011 | 2012 | 2013 |  | 2007 | 2008 | 2009 | 2010 | 2011 | 2012 | 2013 |  |  |  |  |
| N_0_^#^ | 70.7 | 82.4 | 70.9 | 39.5 | 62.5 | 72.9 | 96.6 |  | 73.4 | 114.6 | 111.2 | 114.8 | 98.9 | 76.9 | 100.4 |  | 1186 |  | 169 |
| N_0_+S^#^ | 45.1 | 98.4 | 66.5 | 66.6 | 50.7 | 57.5 | 87.3 |  | 103.6 | 86.2 | 110.4 | 103.1 | 104.0 | 113.3 | 108.2 |  | 1201 |  | 172 |
| N_opt_^§^ | -0.7 | 111.5 | 27.0 | 33.3 | 193.6 | -62.8 | 3.5 |  | -43.9 | 49.8 | -50.2 | -45.4 | 109.7 | 160.0 | 71.2 |  | 557 |  | 80 |
| N_opt_+S^§^ | 85.9 | 153.7 | 34.5 | 93.5 | 160.6 | -56.9 | -76 |  | 30.0 | 1.0 | -44.4 | -138.8 | 146.3 | 64.9 | 37.7 |  | 492 |  | 70 |
| N_con_^§^ | 12.0 | 250 | 231.8 | 49.8 | 485.1 | -364.9 | 59.0 |  | 140.6 | 49.8 | -191.2 | 52.6 | 502.3 | 643.5 | -106.5 |  | 1968 |  | 281 |
| N_con_+S^§^ | 74.8 | 305.9 | 189.1 | 10.8 | 484.1 | 6.4 | 186.2 |  | -68.5 | 235.4 | -96.1 | 57.7 | 294.7 | 311.1 | -90.9 |  | 1901 |  | 272 |
| M_bal_+S | 31.5 | 225.8 | 131.4 | 206.7 | 21.8 | 94.2 | -18.1 |  | -60.9 | -59.6 | -68.7 | 132.8 | -55.1 | 31.5 | -173.6 |  | 440 |  | 63 |
| W_bal_+S | 50.6 | 170.4 | 138.7 | 133.4 | 65.9 | 54.8 | -131.6 |  | 49.7 | 31.5 | -47.9 | 131.3 | 25.7 | -8.4 | -76.1 |  | 588 |  | 84 |

^#^Apparent N mineralization = N_uptake_ + NO_3_-N_post_ – NO_3_-N_previous_ in control treatment.

^§^Apparent N loss = apparent N mineralization + N_fertilization_ + NO_3_-N_previous_ – N_uptake_ – NO_3_-N_post_, where N_uptake_ is the N uptake by the crop aboveground at harvest, NO_3_-N_previous_ is residual NO_3_-N in 0–1 m soil depth after crop harvest, and NO_3_-N_previous_ is residual NO_3_-N in 0–1 m soil depth after the previous crop harvest. The apparent N loss in straw-removal treatments used the value of apparent N mineralization in N_0_ treatment; correspondingly, straw return treatments used the value in N_0_+S treatment. Negative values might be explained by accumulation of NO_3_-N. N balance in 2007-2010 was retrieved from Qiu et al. (2012).

Table S5 Physical properties of the calcareous Fluvo-aquic soil from the field experiment

| Depth (cm) | Particle size (%) | | | Bulk density (g cm^-3^) |
| --- | --- | --- | --- | --- |
|  | Sand | Silt | Clay |  |
| 0-20 | 40 | 32 | 28 | 1.31 |
| 20-40 | 33 | 54 | 23 | 1.47 |
| 40-60 | 37 | 48 | 15 | 1.45 |
| 60-80 | 34 | 46 | 20 | 1.45 |
| 80-100 | 32 | 51 | 17 | 1.45 |
| 100-120 | 24 | 61 | 15 | 1.47 |

Table S6 Soil organic carbon, total nitrogen, nitrate and ammonium in different treatments at 0-20 cm soil depth after the 2010 summer maize harvest

| Treatment | Soil organic carbon  (g C kg^-1^) | Total nitrogen  (g N kg^-1^) | Nitrate  (mg N kg^-1^) | Ammonium  (mg N kg^-1^) |
| --- | --- | --- | --- | --- |
| N_0_ | 7.6±1.4 | 0.91±0.13 | 2.8±1.3 | 0.8±0.3 |
| N_0_+S | 8.2±0.6 | 1.04±0.14 | 3.3±1.7 | 1.0±0.1 |
| N_opt_ | 9.0±2.9 | 1.01±0.08 | 7.7±1.4 | 1.2±0.4 |
| N_opt_+S | 7.9±1.0 | 1.14±0.09 | 8.9±7.4 | 1.1±0.3 |
| N_con_ | 8.4±0.6 | 1.01±0.06 | 23.8±9.3 | 1.5±0.4 |
| N_con_+S | 8.3±0.7 | 1.15±0.07 | 40.7±14.5 | 1.0±0.3 |
| M_bal_+S | 10.2±2.0 | 1.41±0.15 | 7.0±2.7 | 1.1±0.3 |
| W_bal_+S | 9.2±0.3 | 1.37±0.16 | 7.7±1.6 | 1.2±0.1 |

Note: The experiment had started in 2006.


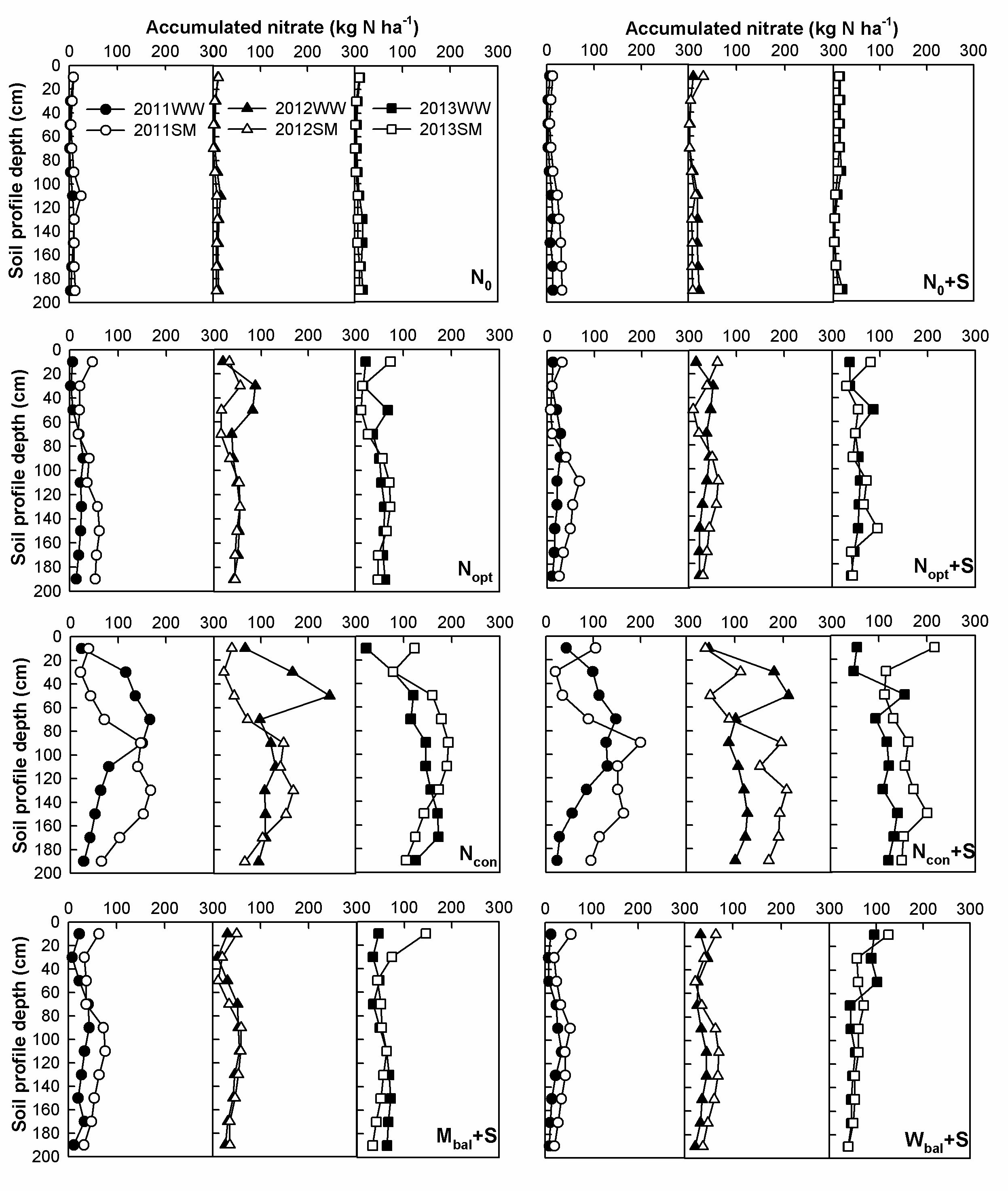


Fig.S1 Soil NO_3_-N (kg N ha^-1^) distribution at 0–2 m soil depth after each crop harvest from October 2010 to September 2013. WW represents winter wheat. SM represents summer maize.

N.B.: N_0_, N_opt_, N_con_, M_bal_ and W_bal_ represent control, improved N_min_ test, conventional farming practice, cattle manure with N balance method and waste compost with N balance method, respectively. S represents straw return.


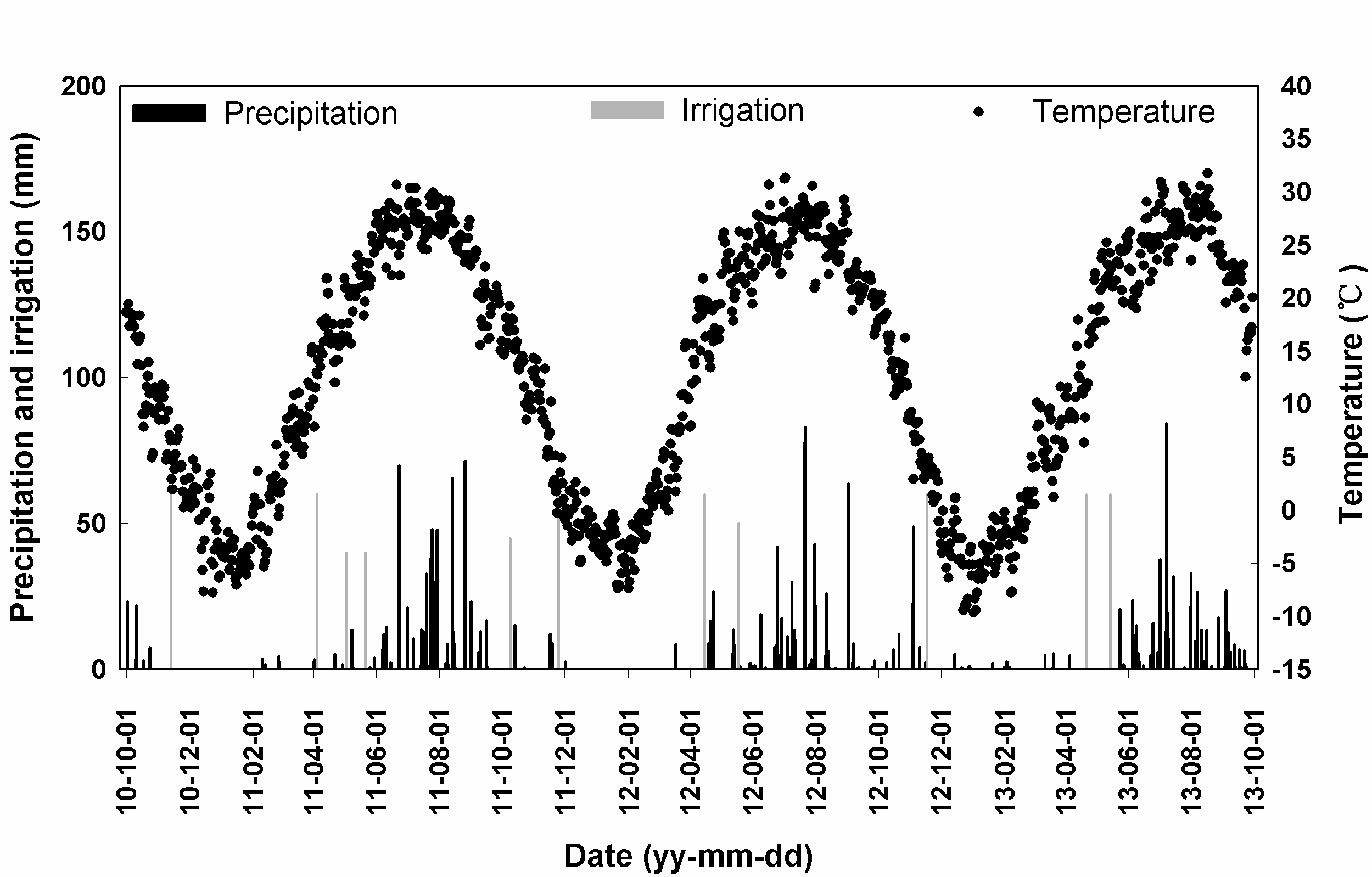


Fig.S2 Air temperature, precipitation and irrigation in the winter wheat-summer maize double cropping system from October 2010 to September 2013.


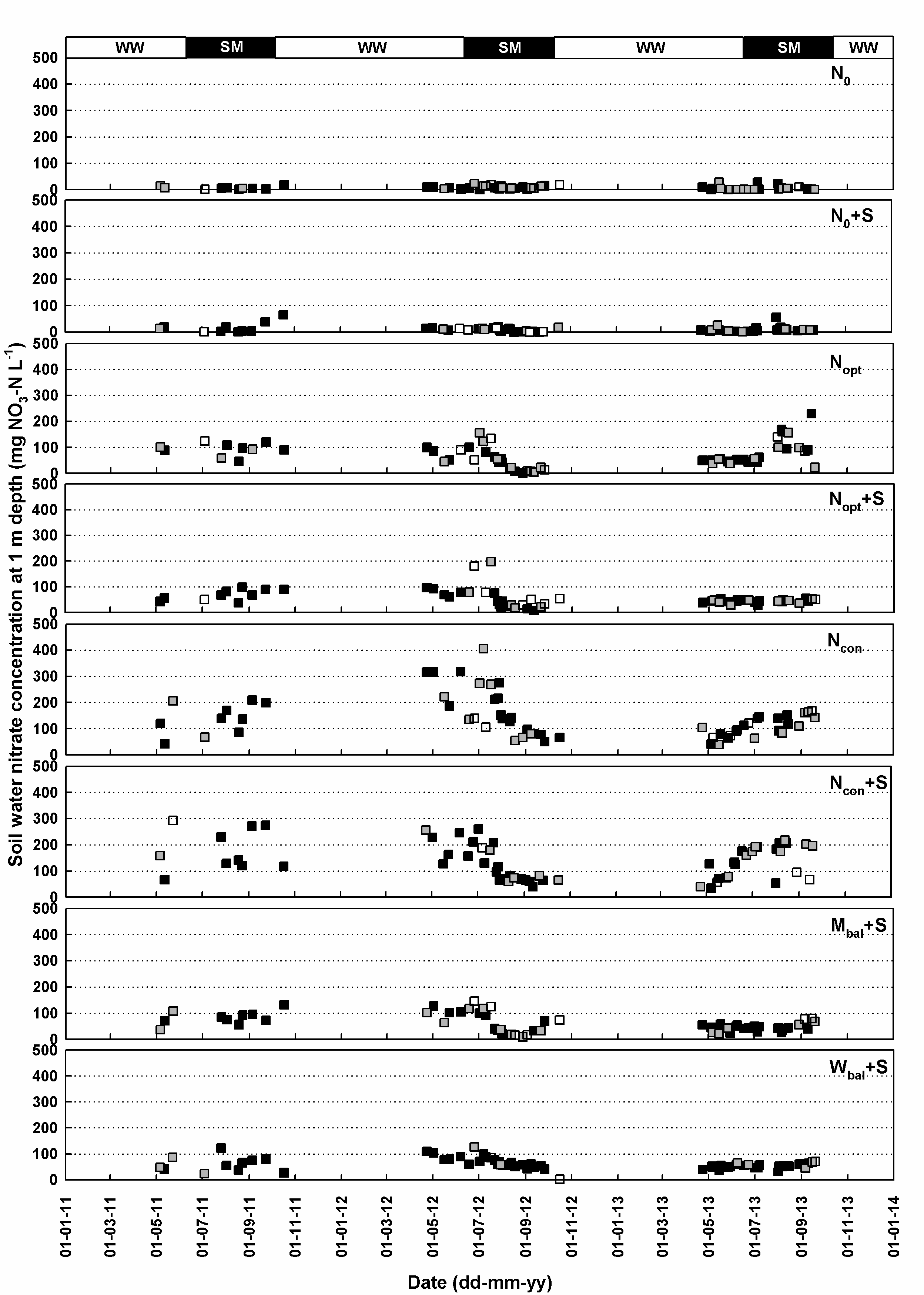


Fig.S3 Soil water NO_3_-N concentrations sampled with suction cups at 1 m depth from January 2011 to December 2013.

N.B.: N_0_, N_opt_, N_con_, M_bal_ and W_bal_ represent control, improved N_min_ test, conventional farming practice, cattle manure with N balance method and waste compost with N balance method, respectively. S represents straw return. The white, gray, and black squares represent soil water samples from one, two, and three replications, respectively.


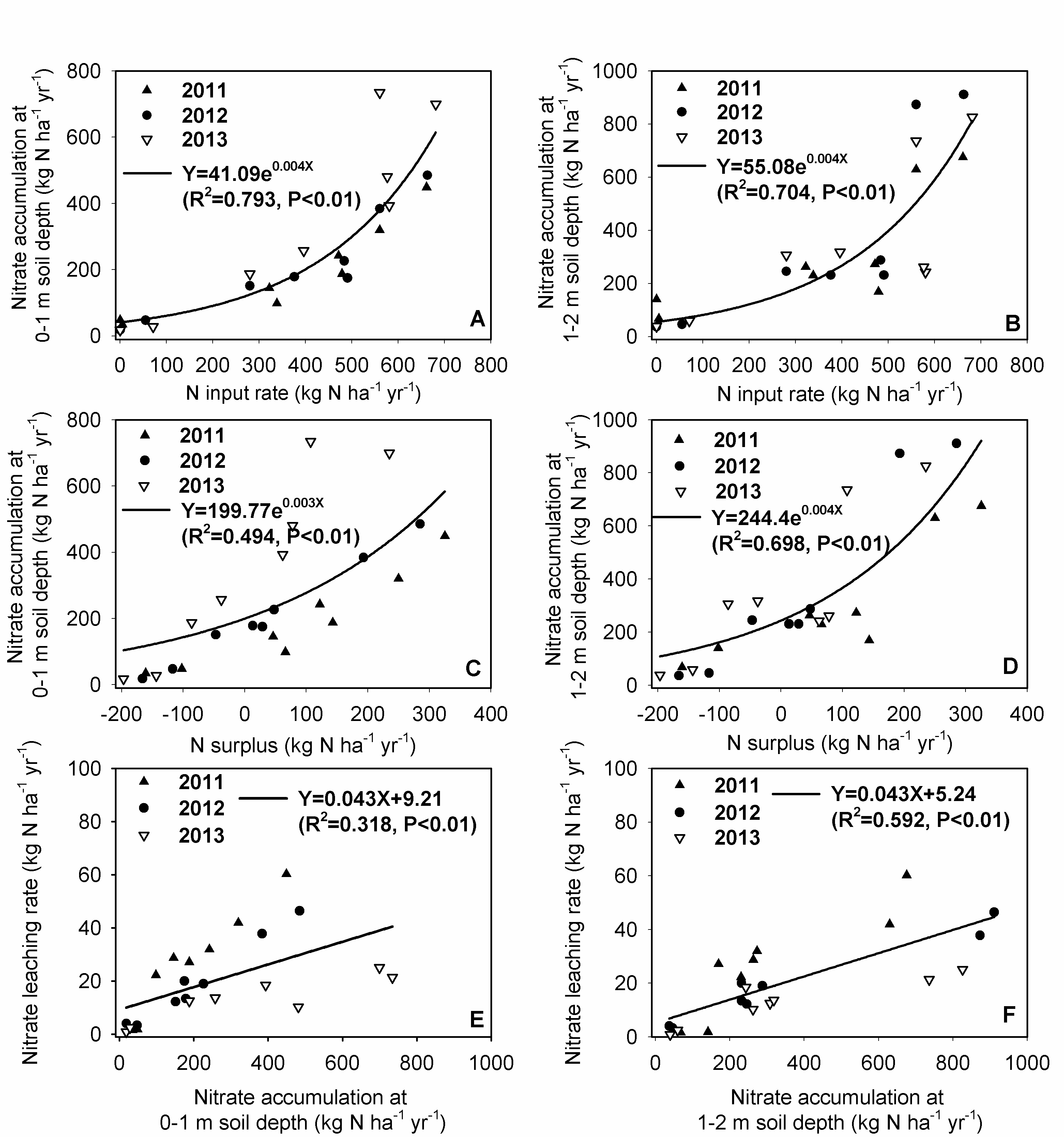


Fig. S4 Correlations between N input and nitrate accumulation at 0-1 m soil depth (A), N input and nitrate accumulation at 1-2 m soil depth (B), N surplus and nitrate accumulation at 0-1 m soil depth (C), N surplus and nitrate accumulation at 1-2 m soil depth (D), nitrate accumulation at 0-1 m soil depth and nitrate leaching rate (E), nitrate accumulation at 1-2 m soil depth and nitrate leaching rate (F).


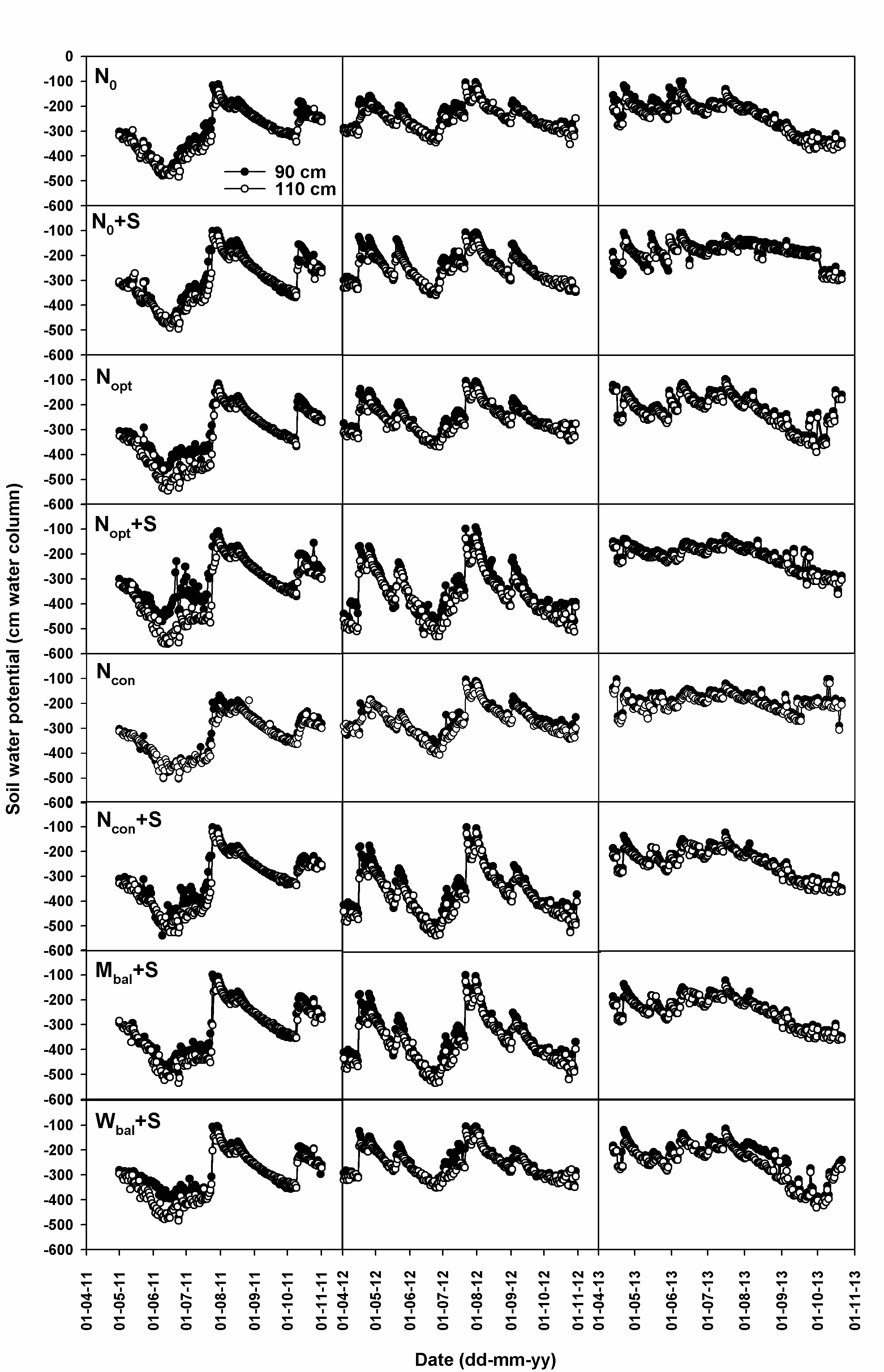


Fig.S5 Soil water potential (cm) at 90 and 110 cm soil depths in the field experiment from April to November in 2010, 2011, and 2012.

N.B.: N_0_, N_opt_, N_con_, M_bal_ and W_bal_ represent control, improved N_min_ test, conventional farming practice, cattle manure with N balance method and waste compost with N balance method, respectively. S represents straw return.


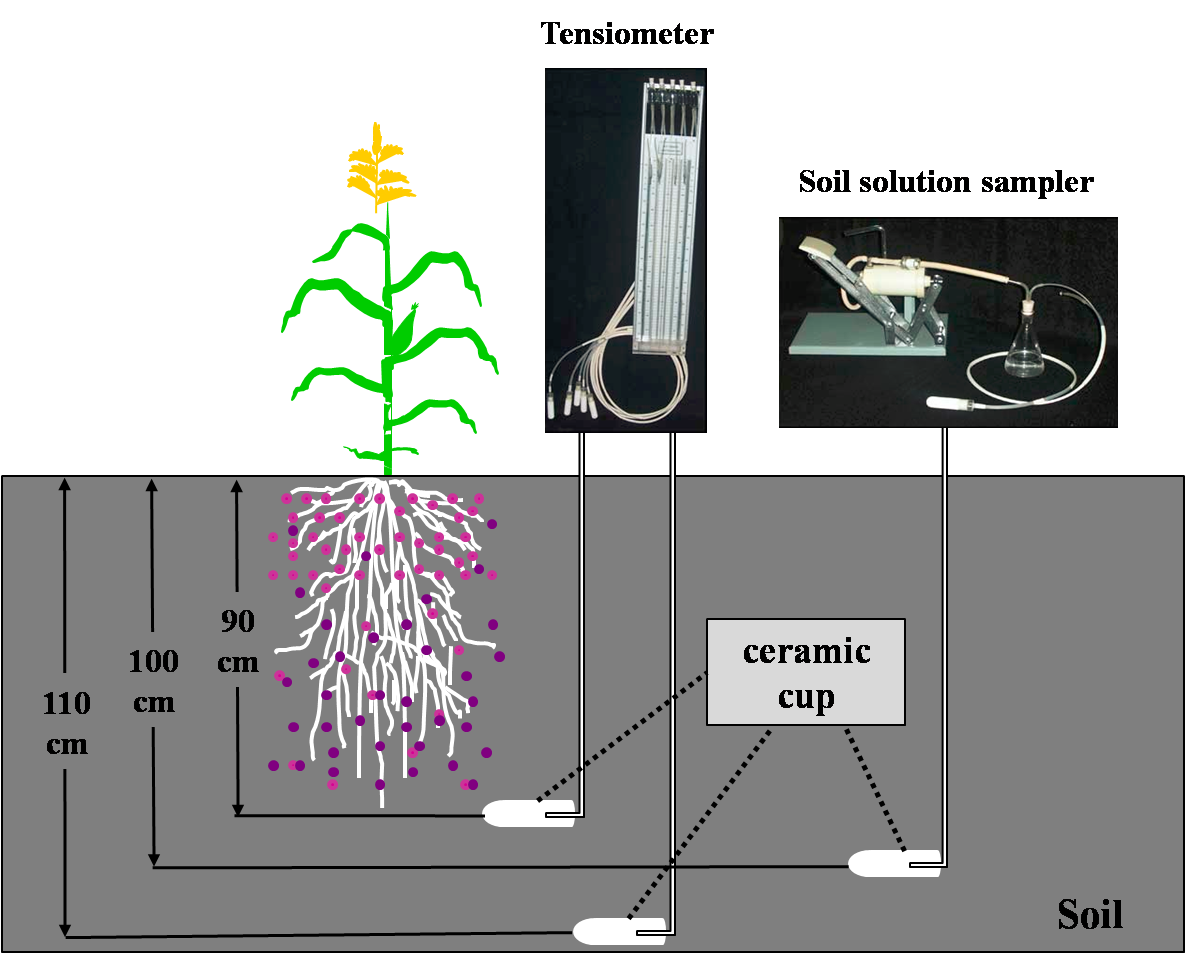


Fig.S6 Schematic view of ceramic cups, tensiometer and soil solution sampler.
